# Computer-Aided Discovery of Small Molecule Inhibitors of Transcriptional Activity of TLX (NR2E1) Nuclear Receptor

**DOI:** 10.3390/molecules23112967

**Published:** 2018-11-14

**Authors:** Evgenia Dueva, Kriti Singh, Anastasia Kalyta, Eric LeBlanc, Paul S. Rennie, Artem Cherkasov

**Affiliations:** Vancouver Prostate Centre, University of British Columbia, 2660 Oak Street, Vancouver, BC V6H 3Z6, Canada; edueva@prostatecentre.com (E.D.); ksingh@prostatecentre.com (K.S.); akalyta@prostatecentre.com (A.K.); eleblanc@prostatecentre.com (E.L.); prennie@prostatecentre.com (P.S.R.)

**Keywords:** TLX, NR2E1, transcriptional factor, nuclear receptor, structure-based drug design, prostate cancer

## Abstract

Orphan nuclear receptor TLX (NR2E1) plays a critical role in the regulation of neural stem cells (NSC) as well as in the development of NSC-derived brain tumors. In the last years, new data have emerged implicating TLX in prostate and breast cancer. Therefore, inhibitors of TLX transcriptional activity may have a significant impact on the treatment of several critical malignancies. However, the TLX protein possesses a non-canonical ligand-binding domain (LBD), which lacks a ligand-binding pocket (conventionally targeted in case of nuclear receptors) that complicates the development of small molecule inhibitors of TLX. Herein, we utilized a rational structure-based design approach to identify small molecules targeting the Atro-box binding site of human TLX LBD. As a result of virtual screening of ~7 million molecular structures, 97 compounds were identified and evaluated in the TLX-responsive luciferase reporter assay. Among those, three chemicals demonstrated 40–50% inhibition of luciferase-detected transcriptional activity of the TLX orphan nuclear receptor at a dose of 35 µM. The identified compounds represent the first class of small molecule inhibitors of TLX transcriptional activity identified via methods of computer-aided drug discovery.

## 1. Introduction

Nuclear receptors (NRs) are a pharmacologically relevant superfamily of transcription factors implicated in numerous human conditions [1]. The activity of many NRs is controlled by steroid hormones or other small lipophilic molecules. However, for a subgroup of nuclear receptors, called orphan receptors, no natural or synthetic ligands have been identified [2].

A representative of an orphan NR family—TLX (or NR2E1) is homolog of a Drosophila tailless protein [3], which is responsible for body segmentation during early embryogenesis [4]. The mammalian TLX is expressed predominantly in the brain and plays an important role in neurogenesis [5,6,7], retina development [8,9], vision [10], and regulation of neural stem cells self-renewal and stemness [11]. TLX is also implicated in the development of gliomas [12,13], neuroblastomas [14,15], and some neurological diseases such as schizophrenia [16,17]. Little is known about the function of TLX in other tissues and organs. However, some data exist suggesting a role of TLX in beta cell regulation [18] and its implication in type 2 diabetes mellitus development [19]. In breast cancer, TLX overexpression is associated with ERα-negative tumors, where TLX knockdown inhibits growth and invasive capacity of triple negative breast cancer cell lines [20]. It has also been demonstrated that TLX is upregulated in prostate cancer (PCa) cell lines, prostatospheroids, and tumor xenograft model of castration-resistant prostate cancer VCaP-CRPC [21,22]. TLX was shown to suppress oncogene-induced senescence in PCa cells by direct transcriptional repression of the CDKN1A gene and direct transactivation of SIRT1 [21]. TLX could also induce resistance to androgen-deprivation through direct suppression of AR gene transcription and signaling in PCa cells [23]. Therefore, small molecule inhibitors of TLX could potentially have high therapeutic value in the number of human conditions, including PCa.

**Figure 1:**
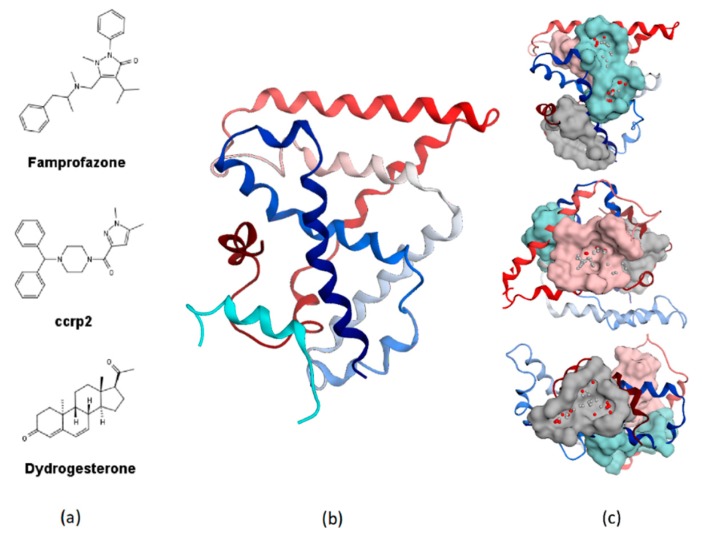
(**a**) Structures of small molecule ligands of TLX, found by medium-throughput screening [30]; (**b**) Structure of TLX LBD in complex with Atro-box peptide (PDB ID 4XAJ) in ribbon representation. TLX is colored from blue for N-terminus to red for C-terminus; Atro-box peptide is colored cyan. (**c**) Surfaces of the pockets in the TLX LBD regions potentially important for transcriptional function: cyan—the region of H1 helix packing, pink—the homodimerization region, grey—Atro-box binding pocket. Spheres represent calculated alpha-spheres that contact 4 protein atoms on its boundary and contains no internal atoms [33].

TLX belongs to the repressor class of orphan NRs and does not recruit conventional NR corepressors such as NCoR and SMRT [9]. It relies on Atrophin [9,24], LSD1 [25], HDACs [26], and BCL11A [27] corepressor for its functional activity. TLX can also act as an activator of gene transcription [21,28,29]. So far, only three compounds (Figure 1a) were found to bind the recombinant TLX LBD [30]. While famprofazone and dydrogesterone represent promiscuous NR binders [31,32], all three compounds potentiated TLX transrepressive activity, while for the therapeutic use this action should probably be reversed [30]. Therefore, further studies are required to identify selective inhibitors of TLX transcriptional activity.

Crystal structures of human and red beetle TLX LBD (Figure 1b) in complex with Atro-box peptide derived from Atrophin revealed auto-repressed conformation of TLX with α-helix H12 occupying a canonical coactivator binding groove with H11 folded into a ligand-binding pocket [34]. This auto-repressed conformation of H12 allows the formation of a previously undescribed binding pocket, which can accommodate the conserved Atro-box motif ALXXLXXY [24]. This conformation is typical for other orphan NRs (DAX-1, SHP, PNR, COUP-TF2, and TR4), and pocket residues contacting Atro-box peptide are highly conserved among them [34].

In this study, we were the first to perform virtual screening of small molecules by docking of Zinc15 database [35] into the Atro-box binding pocket of human TLX. Following in vitro characterization of the top-ranked hits allowed us to identify three chemicals capable to inhibit TLX transcriptional activity in the μmolar range in a dose-dependent manner.

## 2. Results and Discussion

### 2.1. Virtual Screening

Since the crystal structure of TLX LBD is available in Protein Data Bank [34], we applied a structure-based drug design approach to identify possible ligands for human TLX. The surface of TLX LBD is rather flat and contains no significant buried pockets. For the sake of docking of small molecules, one can consider three regions potentially important for transcriptional function of TLX (Figure 1c): the Atro-box binding pocket, the homodimerization surface, and the region of H1 helix packing, which may serve as another site of co-regulator binding [34]. We selected the Atro-box binding site as possessing a proved functional role [9,24] and being less shallow, thus allowing identification of drug-like small molecule binders [36]. We performed docking of the Zinc15 library of commercially available small drug-like [36] molecules in this pocket, using three docking suites: Glide [37,38], OpenEye Fred [39,40], and ICM [41]. Molecules with the best docking scores and docking root-mean-square deviation of atomic positions between molecule poses from three programs less than 2.5 Å were further prioritized for purchasing. The TLX-responsive luciferase reporter assay was developed to evaluate the effect of the selected 97 compounds and known TLX binders on TLX transcriptional activity (Table S1).

### 2.2. Expression of TLX in PCa Cell Lines

**Figure 2:**
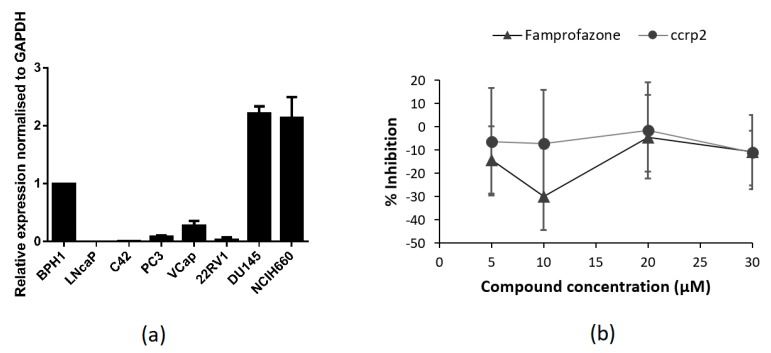
(**a**) Expression levels of TLX in PCa cell lines as measured by qRT-PCR. Error bars represent standard error of mean for two independent experiments performed in triplicates. (**b**) Compounds Famprofazone and ccrp2 known to activate transrepressive activity of TLX did not show dose-dependent inhibitory effect on 3XTAE-LUC reporter activity. Error bars represent standard deviation. Results were obtained from three independent experiments.

To select the cell line for further luciferase-reporter assay development, we analyzed TLX expression levels in a panel of PCa cell lines and in the benign prostatic hyperplasia cell line BPH1 by qRT-PCR. The highest levels of mRNA expression were detected in NCIH660 and DU-145 cells, while the lowest was measured in LNCaP and C42 cells (Figure 2a). The results obtained are in agreement with an earlier work by Wu et al. [21]. In the same work, TLX was shown to directly bind TLX-activating element (TAE) upstream of the SIRT1 promoter and thus activate its transcription [21]. Although further studies are required to show that TAE can be activated exclusively by TLX, TLX binder ccrp2 was able to transactivate SIRT1gene in a dose-dependent manner [21]. Based on these data, DU-145 cells were further transiently transfected with a TLX-responsive luciferase reporter construct pGL3-Basic-3XTAE-LUC, containing three copies of the TAE.

### 2.3. In Vitro Screening (Luciferase Reporter Assay)

The 97 compounds identified during the virtual screening campaign were further tested for their ability to inhibit 3XTAE-LUC reporter activity in DU-145 cells. Six compounds showed greater than 40% inhibition of reporter activity at 35 µM (Table 1). Compound **VPC-33087** capable of inhibiting 100% of reporter activity possessed high cytotoxicity (data not shown). Unlike compound **VPC-33009,** compounds **VPC-33010**, **VPC-33017**, **VPC-33035**, and **VPC-33040** had minimal or no effect on luciferase activity in PC3M cells constitutively expressing the luciferase reporter (Table 1). This provides evidence that compounds **VPC-33010**, **VPC-33017**, **VPC-33035**, and **VPC-33040** inhibit luciferase expression through inhibition of the TLX transcriptional activity rather than through direct binding to luciferase itself. The four compounds were further tested in 3XTAE-LUC reporter assay at two-fold dilution range starting at 25 µM in DU-145 cells. **VPC-33010**, **VPC-33017** and **VPC-33035** inhibited TLX transcriptional activity, as exhibited by a dose dependent inhibition of luciferase expression (Figure S1). For **VPC-33040** the dose response was not significant (Figure S1), higher doses would be required to see an effect, but due to limitations with compound solubility, they could not be tested.

Control compounds famprofazone and ccrp2 were reported to enhance TLX transrepressive activity with EC_50_ values of 9.2 and 1 µM, respectively [30]. In our assay, these compounds did not show dose-dependent effect on 3XTAE-LUC reporter activity (Figure 2b). This observation confirms the distinct mechanism of action of the identified compounds and may indicate that compounds **VPC-33010**, **VPC-33017**, **VPC-33035** and control compounds famprofazone and ccrp2 target different binding sites on the TLX protein.

### 2.4. Binding Mode Analysis

**Figure 3:**
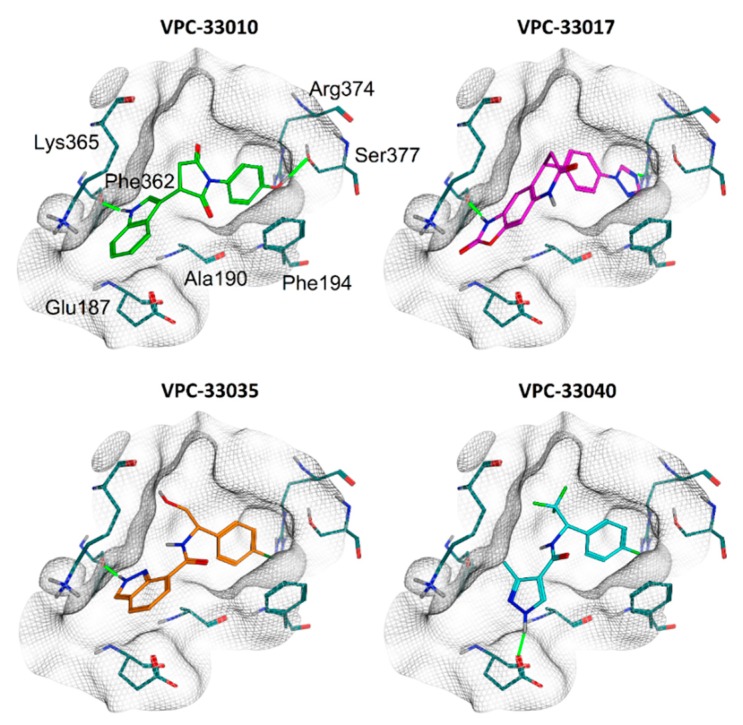
Predicted binding modes of identified compounds in Atro-box binding pocket of TLX LBD. The grids represent pocket surface. Hydrogen bonds are colored green. Only residues forming directed interactions with small molecules are shown.

According to the results of the docking studies compounds, **VPC-33010**, **VPC-33017**, and **VPC-33035** form hydrogen bonds with the backbone carbonyl oxygen of Phe362. In addition, **VPC-33010** and **VPC-33017** form H-bond with Ser377 and Arg374, respectively. In contrast, compound **VPC-33040**, which did not demonstrate dose-dependent effect in the 3XTAE-LUC reporter assay, makes H-bonds with Glu187 (Figure 3). Compounds **VPC-33010** and **VPC-33035** can take part in π–π stacking with Phe194. The compounds’ aromatic rings also form CH–π interactions with aliphatic chains’ hydrogen atoms of Ala190, Lys365, and others. Interestingly all three identified compounds have linear conformation and possess hydrogen bond donors on one or both rims of molecule, which create H-bonds with protein amino acid residues close to the binding site wall (Figure 3). In contrast, among three previously reported TLX binders (Figure 1a), only famprofazone and ccrp2 may serve as H-bond donors at physiological pH; however, their donor groups are located in the central parts of the molecules, which makes the formation of H-bonds on the periphery of the pocket sterically impossible. This also indicates that identified and control compounds target different binding sites. Obtained data may help further informed structure—activity relationship profiling and development of compounds with improved potency.

Our results indicate that Atro-box binding site represents a promising target to regulate TLX activity by small molecules. Identified compounds **VPC-33010**, **VPC-33017**, and **VPC-33035** can inhibit TLX transcriptional activity in the μmolar range. These molecules are the first inhibitors of transcriptional activity of orphan NR TLX obtained from rational structure-based design.This results open new possibilities for further design of TLX inhibitors.

## 3. Materials and Methods

### 3.1. Protein Structure Preparation

The crystal structure of human TLX fused with maltose-binding periplasmic protein with bound Atrophin peptide was extracted from PDB (ID 4XAJ). Fragments corresponding to maltose-binding periplasmic protein and Atrophin were excluded. Chain C was selected for further work as possessing highest geometrical quality. Protein structure was further prepared by protonation and assignment of partial charges in QuickPrep tool from MOE 2016.08022 [33] with default settings and followed by energy minimization in the Amber10 force field [42] to RMS gradient of 0.001 kcal/mol/Å with tethered heavy atoms.

Prepared structure was examined by SiteFinder Tool from MOE 2016.08022 [33] to identify potentially druggable pockets. The Atro-box binding site was then selected as the most suitable for further virtual screening campaign.

### 3.2. Docking

Zinc15 database [35] containing ~7 million of commercially available small organic molecules (molecular weight—250–500, cLogP ≤ 5, availability—in stock) was downloaded 19.06.2017 as energy-minimized 3D structures with protonation corresponding to pH 7.4. PAINs structures as predicted by FAFDrugs4 [43] were excluded.

Docking was performed in three steps [44]: (1) the obtained library was first docked by Glide [37,38] in XP mode in TLX Atro-box binding site. Corresponding receptor grid for docking was prepared in Maestro Version 10.7.0144. (2) Molecules possessing Glide docking score ≤ −6 were prepared for second-round docking by OpenEye docking suite. For every compound, an exhaustive set of conformations was generated with OpenEye Omega2 [45,46]. Docking was performed in FRED [39,40] in Standard precision mode. (3) For compounds with RMSD between Glide and FRED poses less or equal to 2.5 Å final docking was performed in ICM [41]. For a resulting set of compounds with poses RMSD between three docking runs less or equal to 2.5 Å, pK_i_ values were predicted by Scoring.svl [33]. The criteria for final hit selection were highest Glide docking score, pK_i_ value, absence of reactive groups and formation of hydrogen bonds with protein pocket residues. For 156 selected structures, availability and prices were checked and 97 compounds were purchased.

### 3.3. Cell Culture

DU145, LNCaP, PC3M, BPH1, C42, VCaP, 22RV1, NCIH660, and PC3 cells were obtained from ATCC (Manassa, VA, USA). The cell lines were maintained in the following culture media: DU145: Dulbecco’s Modified Eagles Medium (DMEM) (Hyclone, Thermo Fisher Scientific, Waltham, MA, USA) supplemented with 10% Fetal Bovine Serum (FBS); LNCaP, PC3, BPH1,C42, and PC3M: RPMI 1640 supplemented with 5% FBS; NCIH660: RPMI-1640 Medium supplemented with 0.005 mg/mL Insulin, 0.01 mg/mL Transferrin, 30nM Sodium selenite, 10 nM Hydrocortisone, 10 nM beta-estradiol, extra 2 mM L-glutamine, 5% fetal bovine serum; 22RV1: EMEM supplemented with 10% FBS; VCaP: DMEM supplemented with 5% FBS.

### 3.4. Chemicals and Antibodies

Rabbit monoclonal anti-TLX antibody (ab109179) was purchased from AbCam (Cambridge, UK). Compounds were purchased from commercial vendors Asinex (Winston-Salem, NC, USA), ChemBridge (San Diego, CA, USA), Enamine (Kyiv, Ukraine), KeyOrganics (Cornwall, UK), PrincetonBio (Princeton, NJ, USA), UORSY (Kyiv, Ukraine), Vitas-M (Champaign, IL, USA).

### 3.5. Plasmids and Constructs

For the luciferase transcriptional assay, the pGL3-Basic vector was purchased from Promega (Madison, WI, USA). The TLX-responsive luciferase reporter construct, pGL3-Basic-3XTAE-LUC was made by inserting three copies of the TLX-activating element (TAE) after restriction digestion of pGL3-Basic vector with KpnI and HinDIII enzymes.

### 3.6. Transcriptional Assay

TLX-positive DU145 cells and PC3M were seeded on 96-well plates at 4 × 10^3^ cells/well and 10 × 10^3^ cells/well, respectively. After 24 h, the DU145 cells were transfected with 50 ng of the pGL3-Basic-3XTAE-LUC plasmid. After 24 h of transfection, the cells were treated with either test compounds or DMSO. 24 h post-treatment, the cells were lysed with 50 µL of 1× passive lysis buffer (Promega, Madison, WI, USA). Twenty microliters of the lysate from each treatment were transferred onto a white, 96-well, flat-bottomed plate (Corning Life Sciences, Corelle, NY, USA), and the luminescent signal was measured after adding 50 µL of the luciferase assay reagent (Promega, Madison, WI, USA) on a Tecan M200Pro microplate reader (Tecan, Menedorf, Switzerland). Differences in growth were normalised against total protein concentration, which was measured by the bicinchoninic acid (BCA) assay.

### 3.7. Quantitative RT-PCR

Levels of mRNA were analyzed by quantitative RT-PCR (qRT-PCR). For this purpose, cells were seeded onto 10 cm dishes. When the cells were about 80% confluent, RNA was extracted with TRIzol reagent (Life Technologies, Thermo Fisher Scientific) and purified with the RNeasy Mini Kit (QIAGEN, Valencia, CA, USA). The purified mRNA was quantified using a NanoDrop spectrophotometer (NanoDrop, Wilmington, DE, USA). RNA (0.5 μg) was reverse transcribed using the iScript synthesis kit (Bio-Rad Laboratories, Hercules, CA, USA). cDNA product (100 ng) was added to the primer mix. The final concentration of the primers was 5 pM. The sequences of the primers used in the qRT-PCR experiments were as follows: TLX, forward 5′-TTTGGAAGATGCTTGGAGAG-3′ and reverse 5′-TAGGAACGGCTTTGAAAGTG-3′; glyceraldehyde 3-phosphate dehydrogenase (GAPDH), forward 5′-TGCACCACCAACTGCTTAGC-3′ and reverse 5′-GGCATGG ACTGTGGTCATGAG-3′. The fold change in expression of the gene was calculated using the 2^−ΔΔCt^ method with GAPDH as the internal control.

## Figures and Tables

**Table 1:** Compounds showed more than 40% inhibition of 3XTAE-LUC reporter at 35 µM. Results were obtained from three independent experiments and presented as mean ± SD.

Compound ID	Compound Structure	% Inhibition of 3XTAE-LUC Reporter (DU145) at 35 µM	% Inhibition of LUC Reporter (PC3M) at 35 µM
**VPC-33009**	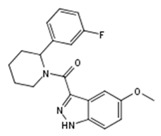	46 ± 9	44 ± 2
**VPC-33010**	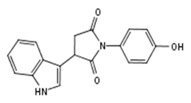	50 ± 9	−9 ± 5
**VPC-33017**	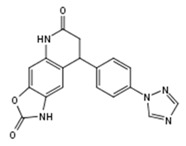	43 ± 13	−2.5 ± 6
**VPC-33035**	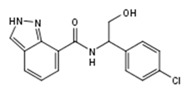	48 ± 12	12 ± 4
**VPC-33040**	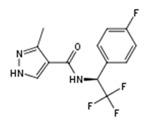	52 ± 17	15 ± 4
**VPC-33087**	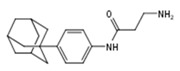	100 ± 0	100 ± 0

## Supplementary Materials

The following are available online, Table S1: Structures of studied compounds and results of reporter activity inhibition measurements at 35 µM in DU-145 cells transfected with TLX-responsive luciferase reporter. Molecular descriptors as calculated in MOE 2016.08022 [33] are also reported. Figure S1: The dose-dependent inhibition of TLX transcriptional activity by lead compounds in DU-145 cells as measured by TLX-driven luciferase reporter assay.
