# Supplementary material for: Computer-Aided Discovery of Small Molecule Inhibitors of Transcriptional Activity of TLX (NR2E1) Nuclear Receptor
Source: Molecules. 2018 Nov 14;23(11):2967. doi: 10.3390/molecules23112967 (PMC6278398; doi:10.3390/molecules23112967)
Supplement: Supplementary file 1 [file molecules-23-02967-s001.zip › Figure_S1.docx]

**Supplementary Figure SI**

**Figure 1:** Dose response curves of the hit compounds. DU145 cells were transfected with the 3XTAE-LUC reporter plasmid and treated with the test compounds for 24h. Compounds VPC-33010, VPC-33017, and VPC-33035 showed dose dependent inhibition of the luciferase expression (A, B, C) but not VPC-33040 (D). Due to limitations with compound solubility, higher doses of the compounds could not be tested.
